# Clinical features and risk factors of bilateral granulomatous lobular mastitis

**DOI:** 10.1097/MD.0000000000037854

**Published:** 2024-04-26

**Authors:** Tingting Ge, Ping Sun, Xue Feng, Xiang Gao, Shuang Gao, Tangshun Wang, Xiaoguang Shi

**Affiliations:** a Department of General Surgery, Dongzhimen Hospital, Beijing University of Chinese Medicine, Beijing, China; b Beijing University of Chinese Medicine, Beijing, China.

**Keywords:** bilateral, granulomatous lobular mastitis, risk factors

## Abstract

Granulomatous lobular mastitis (GLM) is an idiopathic inflammatory breast disease that tends to recur on the same side. With the accumulation of clinical cases, it has been observed that GLM can also occur contralaterally. Currently, most studies on GLM focus on treatment methods and risk factors for ipsilateral recurrence, and there are few reports on bilateral GLM. The study aimed to summarize the clinical characteristics of patients with bilateral GLM by reviewing their clinical data, and to discuss the risk factors affecting the occurrence of bilateral GLM. A retrospective study of the medical records database of patients with GLM admitted between May 2019 and August 2022 was performed. Patients were divided into bilateral GLM group (bilateral GLM group) and unilateral GLM patients (unilateral GLM group). Demographic and clinical characteristics, treatment, and follow-up were collected and analyzed. In this study, by reviewing the clinical data of 59 cases of bilateral GLM, we found that the median time between the onset of bilateral GLM on both sides was 6.63 (0–18) months. Additionally, because of the simultaneous or interval onset on both sides, the duration of the disease was longer compared to unilateral cases. Regarding the history of external hospital treatment, it was found that about 57.63% of patients with bilateral GLM received 2 or more treatment modalities, with a higher involvement of herbal medicine. Meanwhile, by counting the clinical data of the 2 groups of patients with bilateral GLM and unilateral GLM, it was shown by univariate analysis that fertility, nipple development, absolute CD4 value, and CD4/CD8 ratio were associated with contralateral onset of GLM in both groups, with inverted nipple being an independent risk factor.

## 1. Introduction

Granulomatous Lobular Mastitis (GLM), also known as idiopathic granulomatous mastitis s, is a type of non-puerperal mastitis. It is characterized by a palpable breast mass, which can rapidly progress to abscess formation and rupture. In some cases, patients may also experience accompanying symptoms such as low-grade fever, dry cough, lower limb erythema, and arthritis.^[1]^ GLM was previously considered a rare chronic benign inflammatory breast disease, but its incidence has been rapidly increasing in the past 20 years. As of 2021, the Chinese Society of Breast Surgery has published Clinical Practice Guidelines for patients with non-lactational mastitis.^[2]^ Various therapies such as steroids,^[3]^ immunosuppressants,^[4]^ antibiotics,^[5]^ and surgical excision^[6]^ are the main approaches. The choice of treatment method primarily depends on the clinical physician diagnostic habits and experience. In our hospital, a systematic and standardized treatment process has been developed in the long-term clinical treatment. In the retrospective analysis of 1309 patients who received standardized treatment, the recurrence rate was found to be approximately 3.8%. However, with an increase in the number of cases accumulated, a gradual increase in the number of patients with bilateral morbidity was observed.

Most researchers currently believe that GLM mainly presents as unilateral onset, and bilateral onset of GLM has rarely been reported. Upon searching the database, only a few relevant papers^[7–11]^ were retrieved, describing cases of bilateral GLM. However, these papers primarily consisted of case reports or retrospective studies with a limited number of cases. Bilateral GLM is characterized by more severe symptoms, longer disease duration, and greater difficulty in achieving a cure compared to patients with unilateral onset. The prolonged and challenging nature of the disease causes significant suffering for patients and poses a serious challenge for clinicians. This study aims to review the characteristics of bilateral GLM and explore related risk factors to identify clinical risk factors for contralateral onset of the disease. By doing so, we hope to prevent contralateral onset, reduce patients’ pain, and address clinical challenges.

## 2. Materials and methods

### 2.1. Study participants and ethics

This was a retrospective study conducted at Dongzhimen Hospital of Beijing University of Traditional Chinese Medicine (TCM). The study was approved by the Hospital Ethics Committee (No 2023DZMEC-345-02). We followed the STROBE Checklist requirements for this study. This study reviewed the clinical data of patients with unilateral and bilateral GLM who were diagnosed between May 2019 and August 2022 as pathologically confirmed. All patients were over 18 years of age, had no diagnosis of malignant disease, received standardized treatment in our hospital, had complete case histories such as baseline characteristics, inflammatory markers, estrogen levels, and immune-related indicators and could cooperate with follow-up. Males, patients with insufficient follow-up information, and patients with missing data were also excluded.

### 2.2. Subject

**Figure 1.**
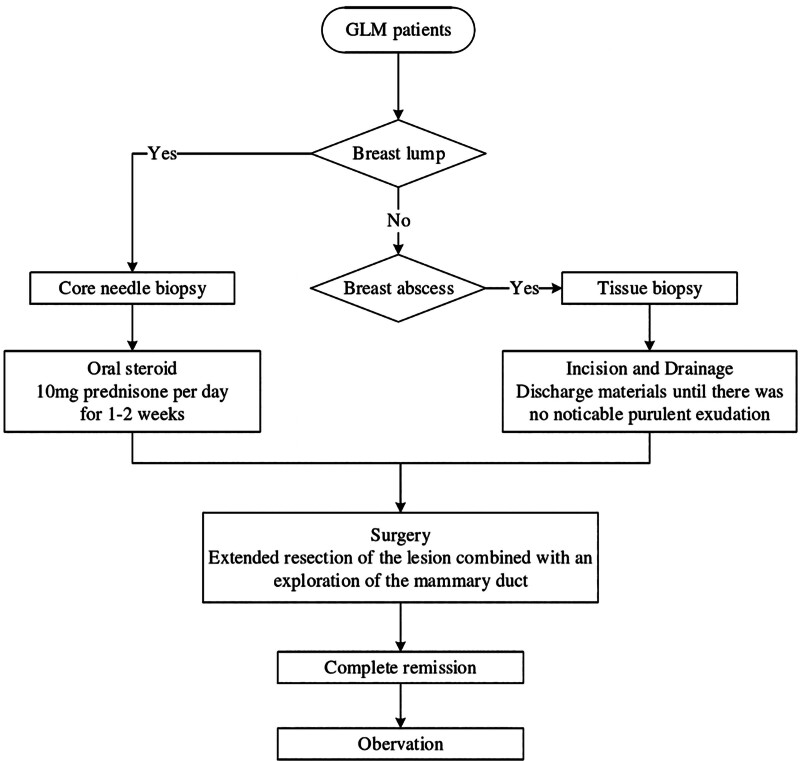
The standard systemic procedure for GLM patients. GLM = granulomatous lobular mastitis.

All patients received a standard treatment regimen at our institution (Fig. 1). If the patient presented with a breast lump as the primary symptom, then core needle aspiration was performed, and after the pathology was clarified, prednisone 10 mg was administered for 7 to 14 days to evaluate the efficacy of the treatment. If the lump did not disappear, surgery was performed. Surgery was performed by mammary duct exploration combined with local resection.

For patients with an abscess stage, tissue biopsy was performed, followed by incision and drainage of the abscess until there was no pus leakage, and then continued with ductal exploration combined with local excision of the lump.

### 2.3. Study data

Patients were followed up for 1 year after consultation in our hospital, with follow-up mainly conducted through by telephone, WeChat, and outpatient clinic. If the patient developed a breast lump on the contralateral side during the follow-up period and was pathologically confirmed as GLM, the patient was defined as a patient with bilateral GLM, and the follow-up was completed. If the patients did not develop contralateral GLM during the follow-up period, they were defined as patients with unilateral GLM. By the end of follow-up, 59 patients with bilateral GLM and 49 patients with unilateral GLM were finally included.

At the follow-up event, the patient general information including age, body mass index, number of pregnancies, history of breastfeeding, nipple development, previous treatments, duration of disease, and treatment modalities were recorded.

The patient laboratory findings, such as platelet count, neutrophil count, lymphocyte count, C-reactive protein, Interleukin 6, Estradiol, Follicle Stimulating Hormone, Luteinizing Hormone, Progesterone, Testosterone, Prolactin, Lymphocyte Subpopulations (CD3, CD4, CD8, CD4/CD8, B-lymphocytes, NK-cells, NKT-cells), antinuclear antibodies, and immunoglobulins (Globulin G/G, Immunoglobulin M (IgM), Immunoglobulin A (IgA), Immunoglobulin E (IgE)), Complement C3, and Complement C4.

### 2.4. Statistical analysis

SPSS (Version 26.0, SPSS Inc., Chicago) was used for statistical analysis. For descriptive analysis of count data, rates, and component ratios were calculated. The chi-square test was used for analysis of variance. When the measurements followed a normal distribution, descriptive analysis was presented using the mean ± standard deviation (𝑥̅±s), and analysis of variance was conducted using the t-test. For non-normally distributed data, descriptive analysis included the median, maximum, and minimum values, with analysis of variance performed using the rank-sum test. Differences in ranked data were assessed using the rank-sum test. Binary logistic regression analysis was utilized for multifactorial analysis of risk factors for bilateral morbidity. A statistical difference was considered when *P* ≤ .05.

## 3. Results

### 3.1. Clinical characteristics of 59 patients with bilateral GLM

Fifty-nine female patients with bilateral GLM were included in this study. The mean age of the patients was 32.53 ± 0.72 years, and none of them had a previous family history of GLM or breast cancer. Fifty-eight patients had a history of fertility, and none of them were pregnant or breastfeeding at the time of diagnosis.

Almost all women diagnosed with bilateral GLM showed a mass with pain, and as the disease progressed, about 79.66% of patients developed progressive abscesses. The abscesses recurred and persisted for a long time, and skin sinus tracts were observed in about 57.63% of the patients. Symptoms such as low-grade fever (8.47%), skin erythema (10.17%), dry cough (10.17%) and joint pain (6.78%) were also observed in a few patients.

In the analysis of the history of outside hospital treatment for bilateral GLM, it was found that approximately 42.37% of patients underwent a single treatment modality, while 57.63% received multiple treatment modalities. The use of TCM in the treatment of GLM was relatively common. Among those receiving a single treatment modality, 23.73% of patients were treated with oral TCM or Chinese patent medicines, and among those who received multiple treatment options, about 18.64% were treated with a combination of TCM and anti-infective therapy. In addition to TCM treatment, a significant proportion of patients also received anti-infective therapy, with 11.86% of patients undergoing single treatment modality receiving such treatment in outside hospitals, and approximately 38.98% of patients undergoing multiple treatment options receiving a combination of anti-infective-based treatment. Further details are provided in Table 1.

**Table 1:** Clinical characteristics of 59 patients with bilateral GLM.

Clinical features	Bilateral GLM (n = 59)
Age at dx [yr, M(range)]	32.53(22~52)
Reproductive history [n (%)]	58(98.3%)
Number of pregnancies [time, M(range)]	1.56(0~5)
Breastfeeding history [n (%)]	55(93.22%)
Breastfeeding duration [mo, M(range)]	12(0~22)
History of mastitis [n (%)]	8(13.56%)
Inverted nipple [n (%)]	24(40.68%)
Bilateral inverted nipple	13(54.17%)
The interval of Bilateral onset [mo, M(range)]	6.63(0~18)
Clinical manifestations	
Pain [n (%)]	59(100%)
Mass [n (%)]	59(100%)
Abscesses [n (%)]	47(79.66%)
Skin sinus tracts [n (%)]	34(57.63%)
Skin erythema [n (%)]	6(10.17%)
Low-grade fever [n (%)]	5(8.47%)
Dry cough [n (%)]	6(10.17%)
Joint pain [n (%)]	4(6.78%)
Treatment methods	
TCM*	14(23.73%)
Anti-infective treatment†	7(11.86%)
Steroids	2(3.39%)
Surgeries‡	2(3.39%)
Anti-infective + TCM	11(18.64%)
Steroids + TCM	3(5.08%)
Surgeries + TCM	4(6.78%)
TCM + Anti-infective + Steroids	2(3.39%)
Anti-infective + Surgeries	6(10.17%)
Anti-infective + Surgeries + Steroids	4(6.78%)
TCM + Surgeries + Steroids	4(6.78%)

GLM = granulomatous lobular mastitis.

* TCM: Traditional Chinese Medicine. In this study, oral Chinese medicines, Chinese Patent Medicines, and external Chinese medicine treatments involving Chinese medicines are all categorized as Traditional Chinese Medicine.

† Anti-infective treatment: Anti-infection includes treatment with antibiotic drugs and anti-tuberculosis drugs.

‡ Surgical options include abscess incision and drainage, lumpectomy, and segmental mastectomy.

### 3.2. Risk factor analysis of patients in both groups

The clinical data statistics revealed a statistically significant difference (*P* ≤ .05) between the 2 groups in terms of parity and inverted nipple. Meanwhile, the clinical laboratory indicators suggested a significant statistical difference (*P* ≤ .05) between the 2 groups in CD4 absolute value and CD4/CD8 ratio (refer to Table 2).

**Table 2:** Clinical factors and medical laboratory indicators between bilateral GLM and unilateral GLM.

Factors	Bilateral GLM(n = 59)	Unilateral GLM(n = 49)	Test statistic	*P* value
Clinical factors				
Age at dx [yr, M(range)]	32.53 ± 0.72	33.15 ± 0.79	t = −0.571	.569
BMI [M(range)]	24.29 ± 2.66	24.13 ± 3.56	t = 0.067	.947
Number of pregnancies [n (%)]	1(1,2)	1(1,2)	Z = −0.554	.580
Reproductive history [n (%)]				
Yes	58(98.3%)	43(87.76%)	*χ* ^2^	.045*
No	1(1.69%)	6(12.24%)		
Duration of breastfeeding [time, M(range)]	12 (6,12)	12 (1.3,18)	Z = −0.752	.452
Lactational mastitis [n (%)]				
Yes	8(13.56%)	6(12.24%)	*χ*^2^ = 0.041	.084
No	51(86.44%)	43(87.76%)		
Inverted nipple [n (%)]				
No	35(59.32%)	38(77.55%)	*χ*^2^ = 4.061	.044*
Yes	24(40.68%)	11(22.45%)		
Bilateral inverted nipples	13(54.17%)	2(27.27%)	*χ*^2^ = 3.988	.046*
Unilateral inverted nipple	11(45.83%)	9(72.73%)		
Medical laboratory indicators				
Neutrophil [×10^9^/L, M(range)]	6.33 ± 2.59	5.80 ± 2.57	t = 0.982	.329
Lymphocyte [×10^9^/L, M(range)]	2.26 ± 2.53	1.85 ± 1.41	t = 0.264	.981
Platelet count [×10^9^/L, M(range)]	296.5(240.25,376.25)	273.00(247.00,338.00)	Z = −0.909	.363
C-reactive protein [mg/L, M(range)]	9.50 ± 2.03	7.50 ± 2.05	t = 0.691	.491
Interleukin 6 [ng/L, M(range)]	7.93 ± 1.31	8.28 ± 1.85	t = −0.156	.876
Estradiol [pg/mL, M(range)]	93.42 ± 8.65	86.68 ± 60.55	t = 0.541	.589
Follicle Stimulating Hormone [mIU/mL, M(range)]	9.44 ± 3.37	5.50 ± 0.50	t = 1.155	.253
Luteinizing Hormone [mIU/mL, M(range)]	9.01 ± 2.35	8.46 ± 2.13	t = 0.173	.863
Progesterone [ng/mL, M(range)]	3.79 ± 0.66	4.13 ± 0.81	t = −0.326	.745
Prolactin [ng/mL, M(range)]	28.71 ± 4.83	22.76 ± 4.86	t = 0.860	.392
Testosterone [ng/mL, M(range)]	0.93 ± 0.57	0.70 ± 0.40	t = 0.324	.747
Complement c1q [mg/L, M(range)]	236.60 ± 5.81	235.35 ± 5.35	t = 0.155	.878
CD3 value [Pieces/µL, M(range)]	1454.04 ± 84.64	1272.24 ± 64.90	t = 1.705	.092
CD4 value [Pieces/µL, M(range)]	851.50 ± 53.76	709.86 ± 36.80	t = 2.714	.032*
CD8 value [Pieces/µL, M(range)]	518.79 ± 32.57	481.35 ± 35.95	t = 0.770	.443
CD4/CD8	1.68(1.36,2.31)	1.49(1.21,1.85)	Z = -2.105	.035*
Immunoglobulins Globulin G [g/L, M(range)]	12.40(11.20,14.40)	12.80(10.10,14.48)	Z = −0.0418	.676
Immunoglobulins Globulin A [g/L, M(range)]	2.30(1.79,2.91)	2.61(1.95,3.71)	Z = -1.294	.196
Immunoglobulins Globulin M [g/L, M(range)]	1.57 ± 0.09	1.59 ± 0.10	t = −0.021	.834
Immunoglobulins Globulin E [g/L, M(range)]	123.39 ± 22.03	70.99 ± 17.99	t = 1.842	.068
Complement C3 [g/L, M(range)]	1.18(0.99,1.37)	1.11(0.92,1.24)	Z = -1.264	.206
Complement C4 [g/L, M(range)]	0.28(0.25,0.345)	0.27(0.21,0.33)	Z = −0.864	.388
Antinuclear antibodies [n (%)]				
Positive	28(47.46%)	18(36.73%)	*χ*^2^ = 1.259	.262
Negative	31(52.54%)	31(63.27%)		

*P < 0.05.

BMI = body mass index, GLM = granulomatous lobular mastitis.

### 3.3. Multiple logistic regression analysis of the 2 patient groups

In this study, we employed multiple logistic regression analysis to evaluate the impacts of parity, inverted nipple, absolute CD4 value, and CD4/CD8 ratio on bilateral GLM. The analysis results revealed that nipple inversion was an independent risk factor for bilateral GLM (OR = 3.496, 95% CI [1.269–9.629], *P* = .015), indicating that patients with nipple inversion were 3.496 times more likely to develop bilateral GLM compared to those without nipple inversion (refer to Table 3).

**Table 3:** Logistic regression analysis of factors of bilateral granulomatous mastitis.

Factors	B	SE	Odds ratio (OR)	95% confidence interval (CI)	*P* value
Parity	−1.778	1.150	0.171	0.018–1.627	.125
Inverted nipple	1.252	0.517	3.496	1.269–9.629	.015
CD4 + T	0.001	0.001	1.001	1.000–1.003	.082
CD4/CD8	−0.069	0.100	0.937	0.769–1.134	.491

## 4. Discussion

The concept of GLM was first proposed in 1972 and named for its pathological manifestation of non-caseating granulomatous inflammation centered around the breast lobules. GLM used to be a rare disease, and although there is currently no large-scale epidemiological evidence to support it, the incidence of GLM has been reported to increase annually both domestically and internationally, particularly in regions such as Turkey and Asia.^[1]^ The diagnosis and treatment of GLM pose significant challenges due to the complex symptoms associated with the condition. Distinguishing GLM from breast cancer is particularly difficult, as both conditions exhibit similar clinical symptoms and radiographic features.^[12]^ Symptoms of GLM primarily manifest as irregular, firm breast masses with poor mobility and may also be accompanied by enlargement of the axillary lymph nodes, often leading to misdiagnosis as malignant breast tumors. On mammography, GLM appears as irregular masses and is frequently classified as BI-RADS 4a to 4c,^[13]^ indicating suspicion of malignancy that necessitates further investigation. Breast ultrasound often reveals heterogeneous, irregular masses with associated skin thickening and enlarged axillary lymph nodes, which can also be confused with the ultrasonographic appearance of breast cancer.^[14]^ Core needle biopsy pathology results are crucial for diagnosis, revealing granulomatous lesions centered around the breast lobules.^[15]^ Due to the similarities in X-ray and ultrasound features with breast cancer, core needle biopsy has become the primary diagnostic method for GLM, boasting a sensitivity of up to 94%.^[16]^ Thus, vigilance is required in the diagnosis and treatment of GLM to avoid misdiagnosis and missed diagnoses. Previous studies have primarily focused on the risk factors and treatment of unilateral GLM, with little mention of the clinical characteristics and risk factors associated with bilateral GLM. This study retrospectively reviewed clinical data from 59 cases of bilateral GLM and found that the median interval between the onset of GLM in both breasts was 6.63 (0–18) months. This provides practical evidence for patient education, suggesting that within 18 months after unilateral GLM, patients should be cautious of risk factors to avoid onset in the contralateral breast. Additionally, because GLM can occur simultaneously or with an interval between the 2 sides, the duration of the disease is longer compared to unilateral cases. In terms of symptoms, bilateral GLM shows similar characteristics to unilateral GLM, although the severity of symptoms was not compared in this retrospective study as the clinical data did not provide such information. Regarding previous treatments received outside the hospital, it was found that approximately 57.63% of patients with bilateral GLM received 2 or more treatment modalities, with a higher involvement of TCM. TCM has unique advantages in the treatment of GLM, as it can reduce the size of the mass and the inflammatory response in the early stage of the disease through treatment based on syndrome differentiation.^[17]^ Combined with surgery, it can significantly shorten the treatment period and improve the surgical cure rate.^[18]^ Overall, TCM has the effect of increasing the cure rate and decreasing the recurrence rate without obvious adverse reactions.

In reviewing reports on risk factors for GLM recurrence in domestic and international studies,^[19–24]^ nipple discharge, skin damage, surgery, breast abscess, and smoking history were commonly identified as independent risk factors for unilateral recurrence (Table 4). This study found statistical differences in 4 variables: fertility, nipple development, CD4 absolute value, and CD4/CD8 ratio, through univariate analysis.

**Table 4:** Risk factors for GLM recurrence reported in the literature.

Author	Patients		Follow-up time [month, M(range)]	Risk factors associated with recurrence
	Recurrence	No recurrence		
Chunxiang Tian^[19]^	125	760	12(3~73)	Bilateral disease [OR 3.036,95% CI:1.753–5.255,*P* = .001] Nipple discharge [OR 3.356,95% CI:1.87–7.100,*P* = .002] Skin lesion [OR 1.663,95% CI: 1.058–2.614,*P* = .028] The therapy of surgery and medication [OR 4.183,95% CI: 2.439–7.175,*P* = .001]
Lermi N^[20]^	57	63	10.26 ± 9.561	Abscess *P *= .001 Surgery *P = *.000
Ciftci Ahmet Burak^[21]^	16	69	39.99 ± 18.93	AGR(Albumin-to-Globulin Ratio) [OR 50.7,95% CI:5.93–434.1,*P* < .001] Smoking [OR 4.45,95% CI:1.04–18.9,*P* = .044]
Azizi A^[22]^	118	356	Not mention	skin lesions [OR = 1.83,95%CI[0.12–3.00] ,*P* = .01]
Qing Ting Tan^[23]^	20	93	132	inflammatory signs and symptoms* Corynebacterium infection*
Pelin Basim^[24]^	20	102	32.5 (19~67)	Vitamin B_12_ [OR 2.78 *P* = .002] Rheumatological disease [OR 4.54 *P* = .004] Complaint—fistula [OR 4.54 *P* = .004] Number of complaints ≥ 3 [OR 2.24 *P* = .012] Multicentricity [OR 2.06 *P* = .002] Erythema nodosum [OR 2.06 *P* = .003] Medical treatment (alone) [OR 4.56 *P* = .002]

GLM = granulomatous lobular mastitis.

* Univariate regression did not show statistical significance.

GLM commonly manifests in women of childbearing age, typically between the ages of 30 and 45, and often occurs within 5 years postpartum. Although the etiology of GLM remains elusive, the association between fertility and disease risk has been substantiated in various studies.^[25]^ Mahmodlou Rahim^[26]^ demonstrated that all 48 patients included in their GLM study had a history of childbirth, with each having breastfed for at least 6 months, thus elucidating a definite correlation between fertility and GLM. Yulong Yin^[14]^ emphasized the potential role of pregnancy and lactation in the occurrence of GLM. Multiple pregnancies and breastfeeding episodes, which augment the permeability of mammary ducts, are deemed high-risk factors for recurrence. The accumulation of milk creates increased ductal pressure and permeability, causing the entry of milk into the lobules and triggering a cascade of immune reactions.

As early as 1972, when GLM was first reported, Kessler and Woollock noted its resemblance to autoimmune diseases such as granulomatous thyroiditis and granulomatous orchitis. In 1979, Brown KL^[27]^ proposed that GLM pathogenesis stemmed from a series of autoimmune reactions triggered by residual milk within the mammary ducts. Subsequent treatment studies^[28]^ demonstrated the efficacy of immunosuppressants and steroid therapy in managing GLM. Through immunohistochemical staining of patient tissues, Erhan Y^[29]^ identified an inflammatory microenvironment primarily composed of T lymphocytes, providing evidence to support the notion of T-cell-mediated immune reactions underlying GLM pathogenesis. Furthermore, our examination of the patient T lymphocyte subpopulations revealed a significant increase in CD4^+^ T lymphocytes in bilateral cases compared to unilateral cases, indicating a statistically significant difference.

In this study, nipple retraction is considered an independent risk factor for recurrent GLM. The etiology of congenital nipple retraction often involves insufficient supporting tissues and inadequate development of the terminal mammary ducts.^[30]^ Due to the narrowing and obstruction of the mammary ducts in patients with nipple retraction, milk cannot be smoothly discharged, resulting in prolonged deposition within the ducts. As the pressure within the ducts increases and causes ductal damage, the overflow of deposits triggers an autoimmune response, thereby initiating the pathological changes associated with GLM.^[31]^

In summary, parity, inverted nipple, absolute CD4 count, and CD4/CD8 ratio are associated with contralateral onset of GLM, with nipple development being an independent risk factor. This provides a cautionary signal for clinicians to closely monitor and follow-up with high-risk individuals to prevent contralateral onset. The main limitation of our study lies in its retrospective analysis and limited data collection, which precluded a comparative analysis of symptoms and severity between unilateral and bilateral cases.

This study still has deficiencies: the study patient group is limited, and the results are biased; the retrospective study may not be comprehensive in determining the clinical symptoms and signs of patients, and there are cases of omission of details, which may lead to bias. A larger randomized controlled study will be conducted subsequently to avoid the deficiencies of retrospective analysis.

## Author contribution

**Conceptualization:** Xiaoguang Shi.

**Data curation:** Tingting Ge.

**Formal analysis:** Tangshun Wang.

**Methodology:** Ping Sun, Xiang Gao, Shuang Gao.

**Resources:** Xue Feng.

**Supervision:** Ping Sun.

**Writing – original draft:** Tingting Ge.
